# Characterization of overwintering sites (*hibernacula*) of the West Nile vector *Culex pipiens* in Central Italy

**DOI:** 10.1186/s13071-025-06710-5

**Published:** 2025-02-24

**Authors:** Federico Romiti, Riccardo Casini, Irene Del Lesto, Adele Magliano, Arianna Ermenegildi, Sarah Droghei, Silvia Tofani, Maria Teresa Scicluna, Verena Pichler, Adriana Augello, Francesco Censi, Paolo Luigi Scaringella, Giovanni Mastrobuoni, Debora Bacciotti, Alberto Nencetti, Claudio De Liberato

**Affiliations:** 1 Istituto Zooprofilattico Sperimentale del Lazio E Della Toscana “M. Aleandri”, Rome, Italy; 2 UniCamillus – Saint Camillus International University of Health Sciences, Rome, Italy; 3 Dipartimento Di Sanità Pubblica E Malattie Infettive, Università Sapienza, Rome, Italy; 4 ASL Latina, Sanità Animale E Igiene Degli Allevamenti, Aprilia, Italy; 5 Soprintendenza Archeologica, Belle Arti e Paesaggio Per Le Province Di Frosinone, Latina e Rieti, Ufficio Territoriale Di Cassino, Cassino, Italy; 6 Via Migliara 45 Dx 34, Pontinia, Italy; 7 USL Toscana Centro - Dipartimento Della Prevenzione, Florence, Italy

**Keywords:** *Culex pipiens*, Diapause, Overwintering, Hibernacula, West Nile virus, Environmental factors, Vector control, Italy

## Abstract

**Background:**

In cool-temperate regions, mosquitoes face winter conditions that hinder their development. To cope with cold temperatures, species like *Culex pipiens*, a major vector of West Nile virus (WNV), diapause as adult females in overwintering shelters known as hibernacula. This study aimed to identify and characterize the overwintering sites of *Cx. pipiens* in central Italy, analyzing the environmental factors influencing the abundance of the two biological forms, *Cx. pipiens pipiens* and *Cx. pipiens molestus*.

**Methods:**

Field surveys were conducted in Lazio and Tuscany over two consecutive winters (2022/2023 and 2023/2024). Overwintering mosquitoes were collected from different hibernacula types, including natural caves, artificial cavities and buildings. Environmental variables such as temperature, humidity, light intensity and vapor pressure deficit (VPD) were recorded in the entrance and inner rooms of each hibernaculum. Mosquito species and *Cx. pipiens* forms were identified through morphological and molecular analyses. A beta regression model was applied to assess the relationship between environmental factors and *Cx. pipiens* abundance. Redundancy analysis (RDA) was used to explore the impact of small- and landscape-scale variables on biological forms distribution.

**Results:**

*Culex pipiens* presence was confirmed in 24 hibernacula and represented the most abundant species, with *Anopheles maculipennis* s.l., *Culiseta annulata* and *Culex hortensis* accounting for 0.4% of the collected individuals. Comparing the entrance and inner rooms, a higher abundance of *Cx. pipiens* s.s. was observed in the darker environments, characterized by a humidity of 50–75%, a temperature of 10–20 °C and a VPD of 0.3–0.8 kPa. Inside the inner rooms, *Cx. pipiens* females preferred lower temperatures, light intensity and humidity, combined with higher VPD. The RDA highlighted that *Cx. pipiens pipiens* was associated with low temperatures and VPD and high humidity levels, preferring semi-natural areas. *Culex pipiens molestus* was positively associated with artificial areas. Hybrids were observed in several types of hibernacula.

**Conclusions:**

This study provides insights into the overwintering ecology of *Cx. pipiens* in southern Europe, highlighting the environmental factors driving its abundance. These results may inform future vector control strategies aimed at reducing mosquito populations and limiting WNV diffusion in temperate regions.

**Graphical Abstract:**

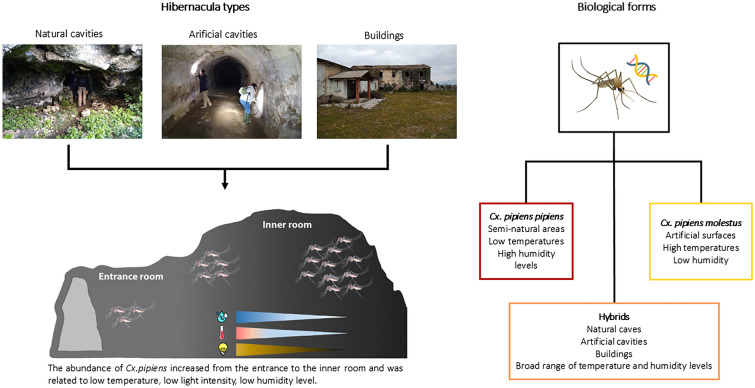

**Supplementary Information:**

The online version contains supplementary material available at 10.1186/s13071-025-06710-5.

## Background

In cool-temperate areas of the northern and southern hemisphere, winter temperatures are a limiting factor for mosquitoes’ development. Depending on the species, mosquitoes can cope with unfavorable environmental conditions by entering a state of dormancy, known as diapause, or by overwintering in a nondiapause state [1]. Diapause may occur at any stage of the life cycle and may consist of the arrest or slowing down of development. Different mosquito genera and species evolved different strategies to survive the winter and ensure population perpetuation, such as the drought-resistant eggs of *Aedes albopcitus* and the cold-tolerant larvae of *Wyeomyia smithii* [1, 2]. Some species within the *Culex*, *Culiseta* and *Anopheles* genera diapause as adult females, seeking dark, humid and protected shelters, referred to as hibernacula (singular hibernaculum), in response to environmental signals.

Mosquito strategies in overcoming the cold season also have a crucial role in the overwintering capacity of mosquito-borne diseases in temperate areas. Considering species that overwinter at the adult stage, diapausing females may be the means by which pathogens survive the winter [3, 4]. Non-blood-fed adult females of some species of the genus *Culex*, sheltered within hibernacula, were found infected with West Nile virus (WNV), Sinbis virus (SINV) and Usutu virus (USUV) in central Europe [5–7]. It has been hypothesized that such positivity may derive from mechanical, vertical or venereal transmission, resulting in overwintering of the virus and early circulation in the next vector season [8].

In Europe, the more epidemiologically relevant vector of WNV is the ubiquitous species *Culex pipiens* [9, 10], with the two biological forms *Cx. pipiens pipiens* (Linnaeus 1758) and *Cx. pipiens molestus* (Forskal 1775), which are morphologically indistinguishable but have different biological traits and genetics [11]. Although both biological forms feed on birds and mammals, *Cx. pipiens pipiens* is considered more ornithophilic and anautogenous and enters diapause during winter [12], whereas *Cx. pipiens molestus* takes blood meals on both birds and mammals; it is autogenous and remains active during winter [13, 14]. Hybrids of *Cx. pipiens pipiens* and *Cx. pipiens molestus* have also been reported under sympatric conditions [15–17], showing a mix of the biological traits of the two forms.

In temperate areas, the last generation of blood-fed *Cx. pipiens pipiens* lay their last batch of eggs in late summer/early autumn. The lower temperatures and a shortened photoperiod experienced by the larvae are the primary environmental cues that trigger the onset of diapause [18]. After larval development and eclosion, the newly emerged adults feed on plant sugar to build up their lipid reserves [19]. Then, females enter hibernation sites without taking a bloodmeal in late autumn/beginning of winter, using the lipids stock during diapause [1, 20, 21].

Hibernacula are generally caves, abandoned buildings or underground sites such as tunnels, basements, water systems, cellars, etc., where female mosquitoes remain almost motionless, attached to surfaces [8, 22, 23]. The choice of hibernacula by mosquitoes is driven by a number of environmental variables, such as temperature and humidity, which remain relatively constant at these sites [24, 25].

Recent studies carried out in central Europe have detected the WNV in diapausing *Cx. pipiens* sampled in their hibernacula [5, 26, 27]. Knowledge of hibernation sites could allow the setting of control strategies aimed at the removal of diapausing adults, a potentially effective control measure [28, 29], considering that the success of diapause has a direct effect on the size of mosquito populations the following season. At present, studies aimed at the identification and characterization of *Cx. pipiens* hibernacula in southern Europe are still limited. In this scenario, we here present the results of a study aimed at investigating *Cx. pipiens* hibernacula in central Italy.

## Methods

### Field activities

Adult overwintering mosquitoes were searched for in different kinds of possible hibernacula in the Lazio and Tuscany regions (central Italy) in two successive winters (2022/2023 and 2023/2024). The inspected sites were located in the province of Viterbo, Latina and Rome (Lazio region) and in the province of Florence (Tuscany region). Hibernacula were categorized as natural caves, artificial cavities—excavated in rocks in recent or ancient periods—and buildings. Examples of artificial cavities were cellars, animal shelters, Etruscan tombs and Roman cisterns. Buildings consisted of abandoned country houses or ground floor premises of hospitals and care homes (Table 1). Each site was divided into two main environments: the entrance and the inner room. The entrance of the hibernaculum is defined as the area exposed (for at least part of the day) to direct sunlight, where the temperature is variable [30]; the inner room is characterized by almost complete darkness, a constant temperature and high humidity [31]. Some of the hibernacula had multiple inner rooms. Light intensity, temperature, humidity and vapor pressure deficit (VPD) parameters were recorded for each of the two indoor environments using a digital luxmeter (BTMETER model CA-BT-881 C/D/E) and a Wi-Fi thermohygrometer (Govee).

**Table 1:** List of sites where at least one hibernaculum was identified, with the number of hibernacula inspected within each site

	Province	Municipality	Site ID	Hibernalucum type	Latitude	Longitude	No. of inspected hibernacula (no. of inner rooms)	Standardized *Culex pipiens* sampling	Environmental variables collection
Tuscany	Florence	Castelfiorentino	FI 1	Building	43.61	10.96	#1 (1)	✔	✔
		Bagno a Ripoli	FI 2	Building	43.73	11.31	#1 (1) #2 (1) #3 (1)	✔	✔
Lazio	Viterbo	Tarquinia	VT 1	Building	42.26	11.75	#1 (3)	✔	✔
			VT 2	Building	42.27	11.78	#1 (1)	✔	✔
	Rome	Rome	RM 1	Building	41.87	12.43	#1 (na)		
			RM 2	Natural cave	41.82	12.50	#1 (1)		
			RM 3	Artificial cavity	41.82	12.50	#1 (1)		
		Nemi	RM 4	Artificial cavity	41.72	12.70	#1 (1)	✔	✔
			RM 5	Building	41.72	12.71	#1 (1)	✔	✔
		Cerveteri	RM 6	Artificial cavity	42.01	12.10	#1 (2) #2 (3) #3 (1)	✔	✔
		Castel Gandolfo	RM 7	Artificial cavity	41.74	12.69	#1 (1) #2 (1)		
	Latina	San Felice Circeo	LT 1	Natural cave	41.22	13.08	#1 (2)	✔	✔
		Sabaudia	LT 2	Artificial cavity	41.26	13.04	#1 (2)	✔	✔
		Norma	LT 3	Building	41.59	12.95	#1 (1)	✔	✔
		Monte San Biagio	LT 4	Natural cave	41.34	13.32	#1 (2)	✔	✔
			LT 5	Natural cave	41.34	13.34	#1 (1)	✔	✔
		Cori	LT 6	Natural cave	41.65	12.93	#1 (2)	✔	✔
		Cisterna di Latina	LT 7	Natural cave	41.59	12.86	#1 (na)		
			LT 8	Artificial cavity	41.59	12.90	#1 (1)	✔	✔
			LT 9	Artificial cavity	41.63	12.84	#1 (1) #2 (1)		
			LT 10	Artificial cavity	41.64	12.85	#1 (1) #2 (1)	✔	✔
			LT 11	Building	41.55	12.80	#1 (1) #2 (1)	✔	✔
		Aprilia	LT 12	Artificial cavity	41.58	12.60	#1 (3)	✔	✔
			LT 13	Artificial cavity	41.63	12.57	#1 (2)	✔	✔

Whenever possible, the standardized sampling method for the quantification of *Cx. pipiens* s.s. was applied, and environmental variables of the inner and entrance rooms were recorded

Adult diapausing mosquitoes detected in the hibernacula were sampled using a portable entomological aspirator. The search and the aspiration were carried out for 1 h during each survey, involving 1–3 operators. To allow a quantitative comparison of *Cx. pipiens* s.s. abundance between the examined hibernacula, the sampling effort was standardized by calculating the mean number of *Cx. pipiens* s.s. collected by an operator in 5 min in both the entrance and each inner room.

### Identification of mosquito species and *Cx. pipiens* biological forms

Sampled mosquitoes were stored at − 20 °C for 30 min to ensure their death and then sorted and identified following the morphological key for the Italian Culicidae fauna [32]. A subsample of *Cx. pipiens* from eight overwintering sites (Table 2) were sent to the Institue of Parasitology of the University of Rome “Sapienza” for molecular characterization to discriminate between the two biological forms *Cx. p. pipiens* and *Cx. p. molestus* and, possibly, detect the presence of hybrids. Genomic DNA was extracted from the whole mosquitoes individually using the DNAzol (MRC Inc., Cincinnati, OH) reagent following the manufacturer’s instructions. Identification of biological forms was performed according to the protocol described by Bahnck and Fonseca [33], and PCR products were visualized by electrophoresis on a 2% agarose gel stained with Midori Green Advance (Nippon Genetics, Tokyo, Japan) and visualized under UV light.

**Table 2:** List of the overwintering sites selected for the molecular analyses of *Culex pipiens* biological forms, with reported results from molecular analysis, the environmental variables recorded in the inner room and the percentage of artificial surfaces, agricultural areas and forest and semi-natural areas in a 2-km radius around each overwintering site

Site ID	*Culex pipiens* biological form: no. (% excluding NA)					Hibernaculum: inner room				Landscape variable (radius 2 km)		
	*Cx. pipiens molestus*	*Cx. pipiens pipiens*	*Cx. pipiens pipiens/molestus*	NA	Total	Temperature (°C)	Humidity (%)	VPD (kPa)	Log_10_(Light + 1) (Lux)	Artificial surfaces (%)	Agricultural areas (%)	Forest and semi-natural areas (%)
FI 1	12 (48.00%)	2 (8.00%)	11 (44.00%)	0	25	22.10	55.00	1.20	0.02	20.09	79.85	0.06
FI 2	12 (70.59%)	0 (0.00%)	5 (29.41%)	0	17	20.67	62.10	0.94	3.38	19.51	79.27	1.22
RM 6	0 (0.00%)	8 (61.54%)	5 (38.46%)	7	20	9.88	75.07	0.30	5.01	9.67	84.24	6.09
LT 2	4 (13.79%)	24 (82.76%)	1 (3.45%)	10	39	12.56	73.34	0.41	0.14	3.43	33.53	21.41
LT 10	3 (7.89%)	26 (68.42%)	9 (23.68%)	2	40	13.30	71.65	0.44	2.97	0.00	81.80	18.20
LT 11	0 (0.00%)	14 (73.68%)	5 (26.32%)	5	24	14.83	50.13	0.85	6.03	2.94	97.06	0.00
LT 12	9 (16.67%)	40 (74.07%)	5 (9.26%)	15	69	13.34	64.56	0.53	8.92	1.76	96.34	1.90
LT 13	0 (0.00%)	22 (84.62%)	4 (15.38%)	0	26	12.95	75.66	0.37	0.58	4.52	95.48	0.00

### Statistical analysis

#### Hibernacula’s environmental characterization

A subset of 15 sites was selected to characterize the two environments of the hibernaculum: the entrance and the inner room. The criteria for site selection included the identification of both an entrance and one or more inner rooms in which it was possible to quantify the *Cx. pipiens* s.s. specimens collected over a 5-min period and record environmental variables. In fact, access to some inner rooms was restricted in certain situations, such as in narrower caves, or, conversely, in healthcare facilities, standard sampling in areas identified as “entrances” was hindered by the presence of personnel. For sites inspected multiple times, or those with more than one inner room and entrance (e.g. a group of country houses on the same site), the average environmental variables and the mean number of *Cx. pipiens* s.s. collected over 5 min were calculated separately for each environment (i.e. entrance and inner room). The percentage of *Cx. pipiens* s.s. collected at the entrance and within the inner room was then computed for each site. Descriptive statistics, including means, medians, standard deviations (SD) and interquartile range (IQR), were calculated for light exposure, temperature, humidity, VPD and the percentage of *Cx. pipiens* s.s. collected across the two environments. To assess whether significant differences existed in the recorded values of light exposure, temperature, humidity, VPD and the percentage of *Cx. pipiens* s.s. collected between the entrance and inner room, a Wilcoxon rank-sum test was performed.

#### *Culex pipiens* s.s. abundance in inner rooms

Considering the preference of *Cx. pipiens* adult females for the dark zones of the hibernacula, which provide a relatively mild and constant climate [34], the following analyses focused on the inner rooms. The relationship between the abundance of *Cx. pipiens* s.s. and light, VPD, temperature and humidity was investigated using data from the inner rooms of 18 sites, selecting those where standardized mosquito sampling was carried out and environmental variables were recorded (sites marked in Table 1). As some sites had more than one inner room and/or were inspected more than once, the average number of *Cx. pipiens* s.s. collected in a 5-min period at each site was calculated and used as dependent variable (y). These averages have been linearly transformed from their original scale to range in the unit interval (0, 1) by applying the following formula:

$${y}{\prime}=\frac{y-a}{b-a}$$

where *a* and *b* are the minimum and maximum number of *Cx. pipiens* s.s. collected over a 5-min period, respectively. Then, the *Cx. pipiens* s.s. abundance index (A_i_) was calculated, compressing *y’* to avoid zeros and ones by taking:

$${A}_{i}=\left[{y}{\prime}\times \left(N-1\right)+ \frac{1}{2}\right]\times \frac{1}{N}$$

where *N* is the number of surveyed sites, as suggested by Smithson and Verkuilen [35]. A beta regression model was performed using the ‘betareg’ function in R [36] (Cribari-Neto and Zeileis, 2010), with *A*_*i*_ as the dependent variable and environmental factors and hibernaculum category as independent variables. The hibernaculum category was included as a three-level categorical variable, dividing hibernacula into artificial cavities, natural caves and buildings. To examine differences in *A*_*i*_ among categories, an artificial cavity was considered as reference category, calculating the odd ratios for buildings and natural caves. The changes in the log odds (*β* coefficients) of the mean response variable relative to the reference category were used to calculate odds ratios as follows: exp^(*β*Building)^ and exp^(*β*Natural cave)^. To reduce the skewness of light intensity data, a log(*x* + 1) transformation was applied. The model was fitted using maximum likelihood estimation, and the precision parameter (*ϕ*) was modeled with an identity link function. To account for the variability in sample size between sites, the total number of *Cx. pipiens* s.s. collected in the inner room(s) during the 5-min samplings was calculated per each site. This site-dependent count was used as a weight parameter in the beta regression analysis.

#### RDA analysis

Two redundancy analyses (RDAs) were performed to explore the relationship between small- and landscape-scale environmental variables (predictors) and the relative abundance of the biological forms of *Cx. pipiens* (response variables). The objective was to define which predictors explain most of the variance of the response variable, indicating their effect in terms of magnitude and direction. The results from the molecular analysis performed to identify *Cx. pipiens* biological forms were used to calculate the percentage of *Cx. pipiens pipiens*, *Cx. pipiens molestus* and *Cx. pipiens pipiens/molestus* in the inner rooms of eight sampling sites. Averages of temperature, humidity, VPD and light intensity (log transformed) were used as small-scale environmental predictors. To characterize the landscape of each site, land cover data were obtained from the Copernicus catalogue [37] by downloading the latest version of the Corine Land Cover (CLC) vector-based dataset. The first level of the CLC categories was considered, calculating the percentage of artificial surfaces (CLC.1), agricultural areas (CLC.2) and forest and semi-natural areas (CLC.3) in a circular buffer around each site. The intersection function in QGIS was used to extract the portions of features from the CLC vector layer that overlap the circular buffers of the overlay layer [38]. The radius of the buffer was set to 2 km considering the dispersal distance of *Culex* mosquitoes [39, 40]. Before performing the RDA, both response and explanatory variables were standardized by scaling (mean = 0, standard deviation = 1) to ensure comparability and to avoid biases caused by differences in measurement scales. The RDA was performed using the vegan package in R [41, 42]. The permutation-based ANOVA (999 permutations) was used to assess the significance of the RDA models, testing whether the explanatory variables collectively explained a significant portion of the variance in *Cx. pipiens* biological form composition.

## Results

### General outcomes

**Fig. 1:**
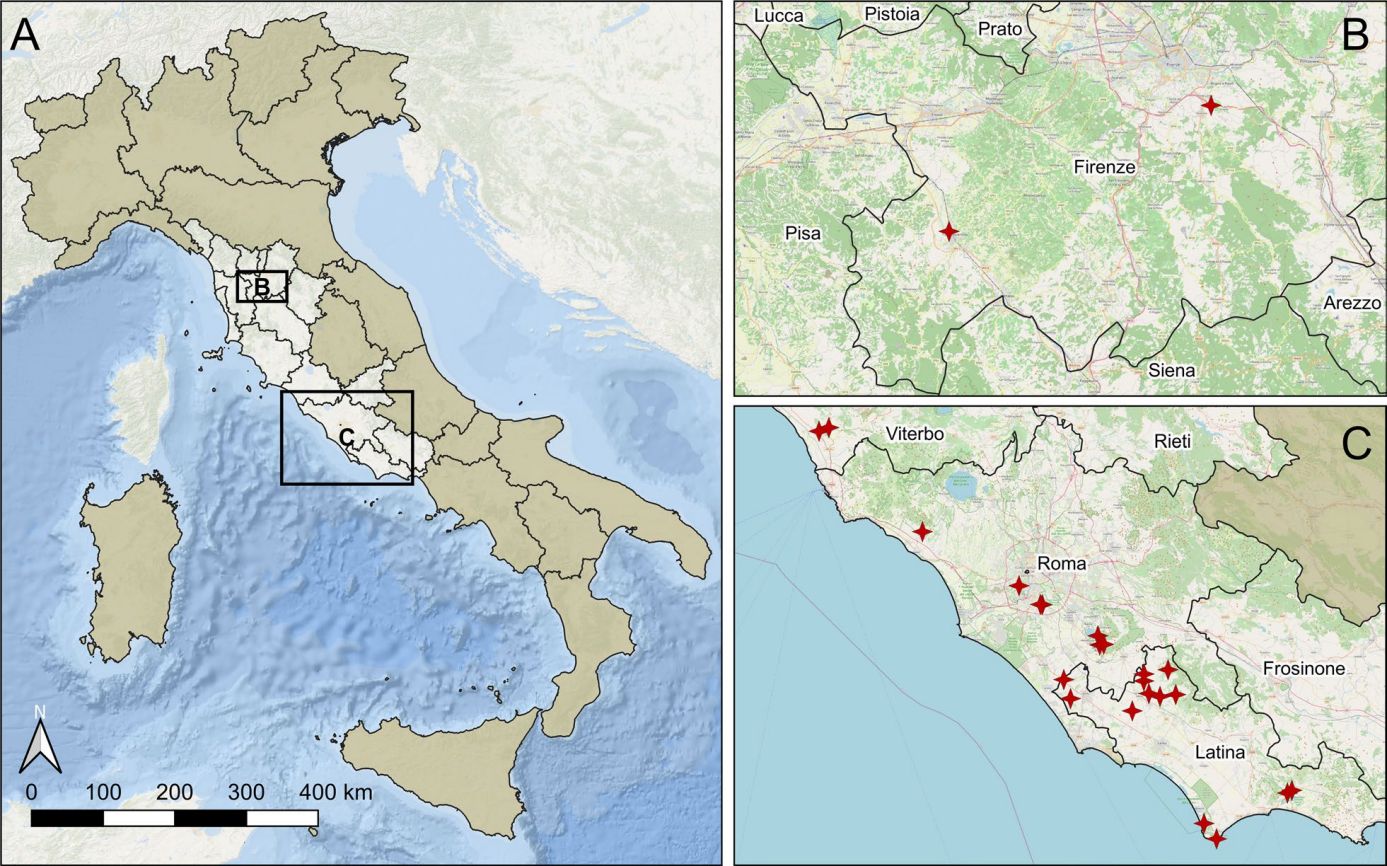
Map of Italy showing the two areas in the regions of Tuscany and Lazio where the research on overwintering sites was carried out (**A**). Zoom-ins on the province of Florence (**B**) and on the area of the provinces of Viterbo, Rome and Latina (**C**), with red crosses indicating the 24 overwintering sites

**Fig. 2:**
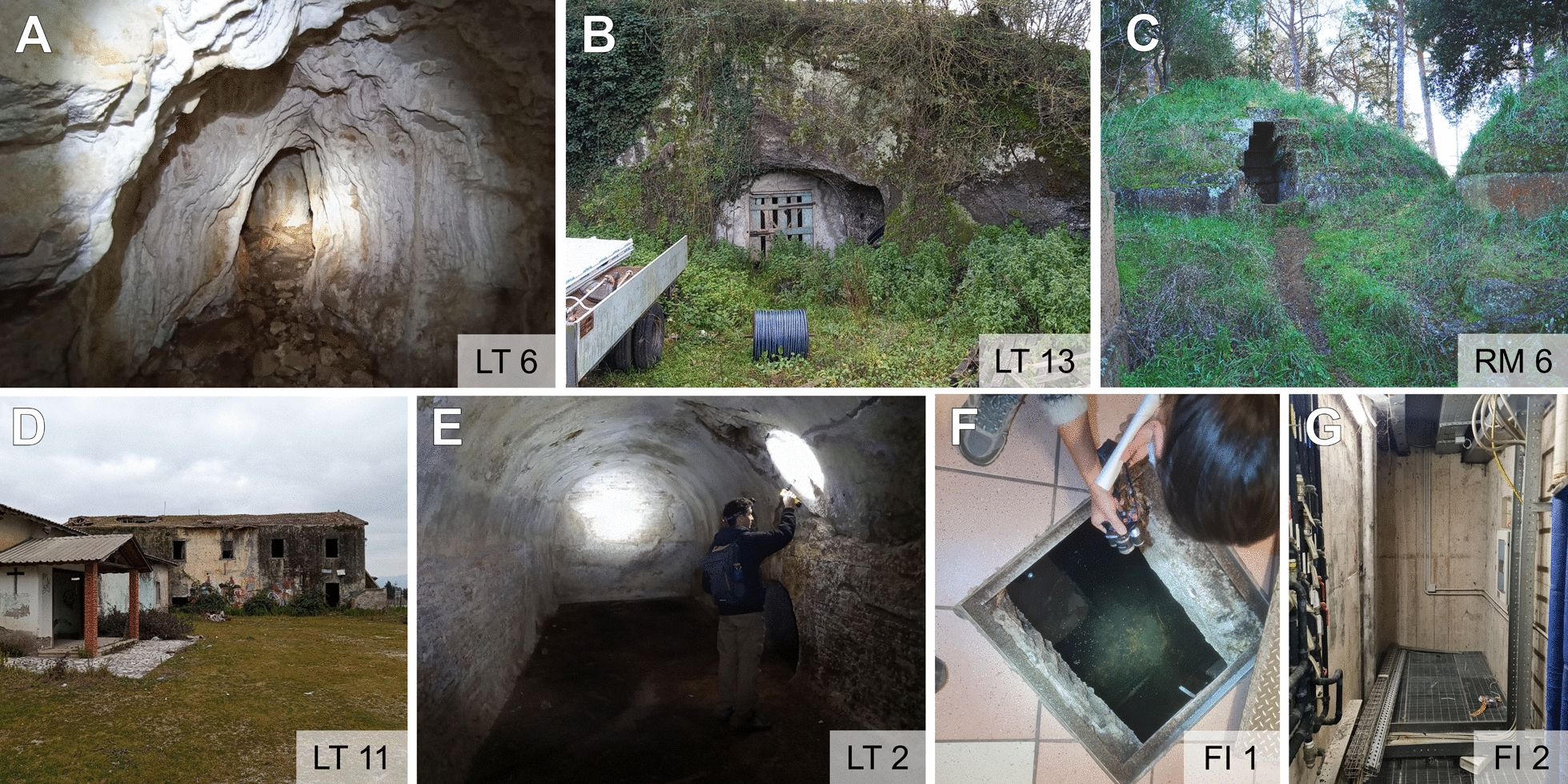
Examples of *Culex pipiens* s.s. hibernacula in the Lazio and Tuscany regions. Natural cave in (**A**) and two artificial cavities dug into tuff rocks in (**B**) and (**C**), an excavated cellar in the hillside and an Etruscan tomb, respectively. A group of abandoned buildings in (**D**), a cistern from the Roman period in (**E**) and two examples of premises in two buildings: the flooded foundations of a care home in (**F**) and a room for cables in a hospital in (**G**)

A total of 10,532 mosquitoes belonging to the following species were collected in 19 hibernacula in Florence, Viterbo, Rome and Latina provinces: *Cx. pipiens* s.s. (*N* = 10,484; 99.54%), *Culex hortensis* (*N* = 36; 0.34%), *Anopheles maculipennis* s.s. (*N* = 8; 0.08%) and *Culiseta annulata* (*N* = 4; 0.04%) (Table 3). Adult females accounted for 99.76% of the collected individuals (*N* = 10,507); *Cx. pipiens* s.s. was detected in all overwintering sites (Fig. 1C). Specimens were caught in natural caves (Fig. 2A), cavities dug into tuff rocks in recent times (Fig. 2B), Etruscan tombs (tenth to second century B.C., Fig. 2C), abandoned country houses (Fig. 2D), Roman cisterns (I century A.D., Fig. 2E) and the ground floor premises of hospitals and care homes (Fig. 2F, G). The largest catches of *Cx. pipiens* s.s. were made in the LT 2 and LT 12 overwintering sites, which were artificial cavities (Table 1). The first one (LT 2) was an underground Roman cistern excavated along Sabaudia Lake, where the resting surfaces for the mosquitoes were plastered walls; the second (LT 12) was an artificial cavity excavated in tuff rock, probably used as a shelter for animals, whose entrance was at ground level. Calibrating captured *Cx. pipiens* s.s. to sampling effort, the following sites reported the highest abundance (> 100 per operator-hour): LT 2, LT 4, LT 8, LT 9, LT 10, LT 11 and LT 12. All these hibernacula were located in rural areas and consisted mainly of artificial cavities, with two exceptions: an abandoned country house (LT 11) and a natural cave (LT 4).

**Table 3:** Summary of the results of mosquitoes collected in the overwintering site with reported number of females and males for each species

Site ID	Culicidae	Samplings no.	*Culex pipiens* s.s. (♀/♂)		*Culex hortensis* (♀/♂)		*Culiseta annulata* ♀	*Anopheles maculipennis* s.l. ♀
FI 1	55	1	54	1	0	0	0	0
FI 2	51	1	47	4	0	0	0	0
VT 1	10	1	10	0	0	0	0	0
VT 2	2	1	2	0	0	0	0	0
RM 4	44	1	44	0	0	0	0	0
RM 5	94	1	87	0	7	0	0	0
RM 6	63	3	62	0	0	0	0	1
LT 1	79	2	79	0	0	0	0	0
LT 2	5264	5	5247	12	0	0	4	1
LT 3	51	2	47	0	4	0	0	0
LT 4	335	2	334	1	0	0	0	0
LT 5	88	2	84	1	0	0	0	3
LT 6	92	2	67	0	24	1	0	0
LT 8	593	2	593	0	0	0	0	0
LT 9	547	1	547	0	0	0	0	0
LT 10	591	2	590	1	0	0	0	0
LT 11	256	2	253	0	0	0	0	3
LT 12	2100	4	2096	4	0	0	0	0
LT 13	217	2	217	0	0	0	0	0
Total	10,532	37	10,460	24	35	1	4	8

### Hibernacula’s environmental characterization

**Fig. 3:**
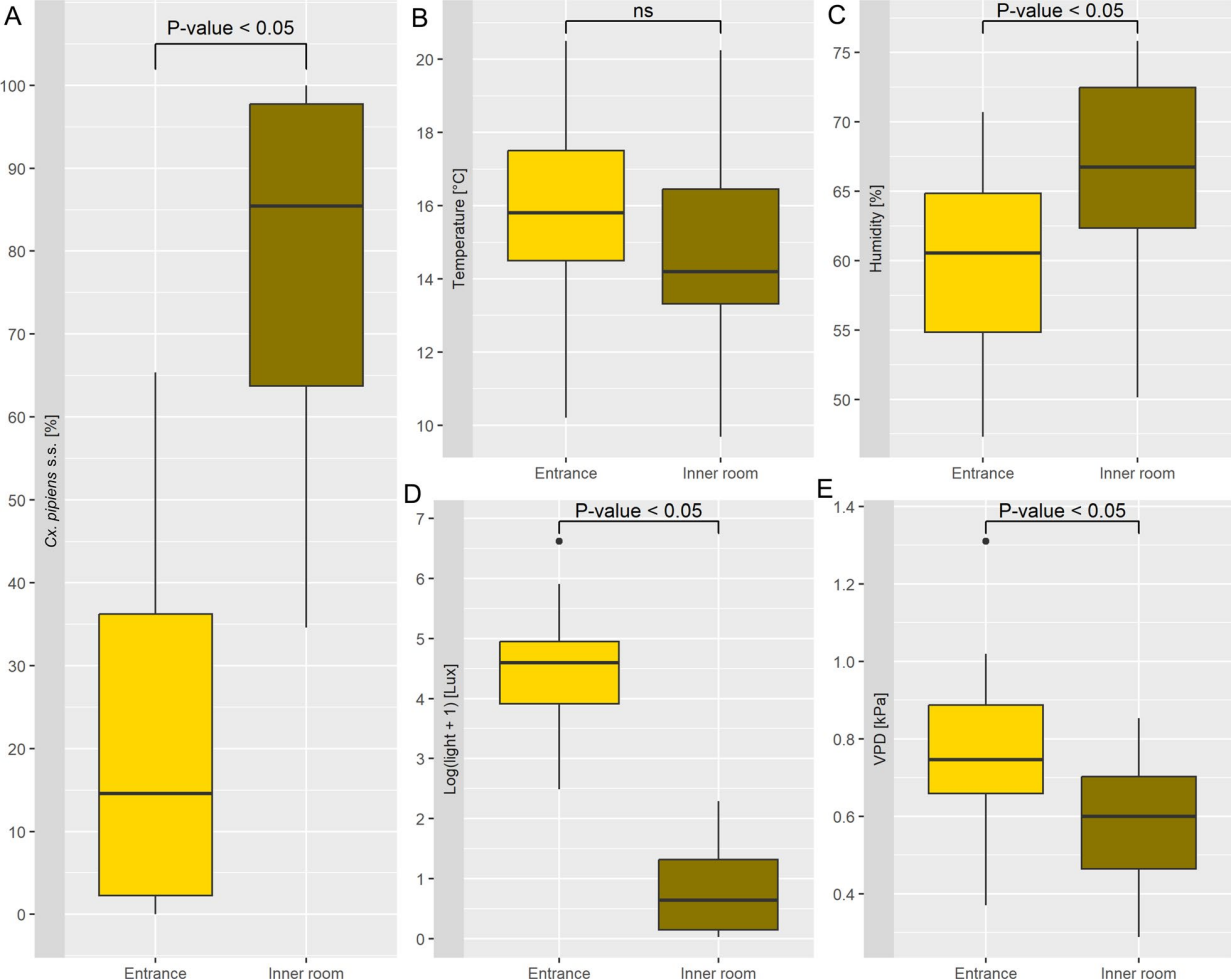
Boxplots for the comparison of *Culex pipiens* s.s. abundance and environmental variables between entrances (yellow) and inner rooms (dark yellow) of the hibernacula. **A** *Culex pipiens* s.s. abundance is reported as percentage of individuals captured in 5 min by a single operator. **B**–**E** Mean values of temperature, relative humidity, light intensity and vapor pressure deficit (VPD) calculated by site/environment. The statistical significance between the two environments (entrance and inner room) is indicated by the *P*-values from Wilcoxon rank-sum tests, where significant differences are marked as *P* < 0.05

The results showed a clear distinction between the entrances and inner rooms of the hibernacula in terms of mosquito presence and environmental variables. The percentage of *Cx. pipiens* s.s. was significantly higher in inner rooms, with a median of 85.4% (IQR: 34.0) compared to a median of 14.6% (IQR: 34.0) at the entrances (Wilcoxon rank-sum: Z = 12, *P* < 0.0001), suggesting that environmental conditions of the inner rooms are more favorable for mosquito presence. The Wilcoxon rank-sum test for humidity showed a significant difference (Z = 60, *P* = 0.03) between the inner rooms (median: 66.7%, IQR: 10.1) and entrances (median: 60.5%, IQR: 10.0). Light levels showed a marked difference, with the inner rooms being darker (median: 0.64 Log[lux + 1], IQR: 1.18) compared to the entrances (median: 4.60 Log[lux + 1], IQR: 1.04). The Wilcoxon rank-sum test confirmed this difference (Z = 225, *P* < 0.0001). Temperature showed a small and not significant difference (Wilcoxon rank-sum test: Z = 147, *P* = 0.16) between inner rooms, slightly cooler (median: 14.2 °C, IQR: 3.13), and entrances (median: 15.8 °C, IQR: 3.01). Finally, VPD was lower in the inner rooms (median: 0.60 kPa, IQR: 0.24) than at the entrances (median: 0.75 kPa, IQR: 0.23), with a significant result of the Wilcoxon rank-sum test (Z = 167, *P* = 0.03). A comparison of microhabitat environmental variables and mosquito abundance within entrances and inner rooms of the hibernacula is reported in Fig. 3.

### *Culex pipiens* s.s. abundance in inner rooms

**Fig. 4:**
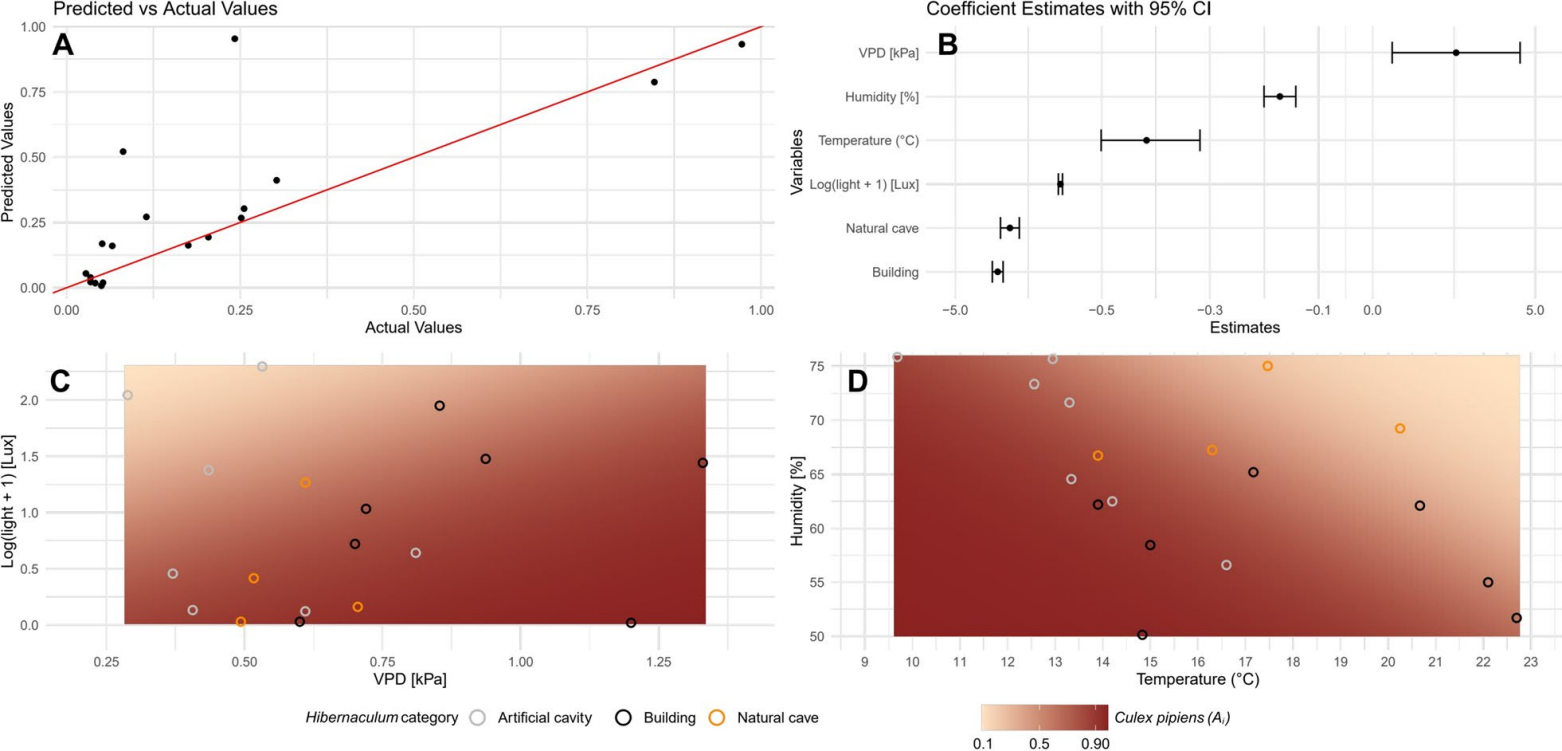
Results of the beta regression model. **A** Scatter plot of actual values of the *Culex pipiens* s.s. abundance index vs. that predicted by the beta regression. The red line indicates perfect prediction; hence, points closer to the line indicate higher prediction accuracy by the model. **B** Coefficient estimates for each of the predictor variables reported on the y-axis. The x-axis shows the magnitude and direction of the effect with error bars representing the 95% confidence intervals. **C** Plot shows the predicted abundance index (*A*_*i*_) of *Cx. pipiens* s.s. as a function of vapor pressure deficit (VPD) and log-transformed light intensity. **D** Predicted *A*_*i*_ was reported as a function of mean temperature and mean humidity. Heatmaps represent the changes in *A*_*i*_, with darker shades indicating higher values. Superimposed points represent observed data, colored by the hibernaculum category

The results of the beta regression model reported a high precision parameter value (*ϕ* = 10.75, *P* < 0.0001) and a low dispersion of the response variable (*A*_*i*_ variance = 0.02). These results indicate that the variability of the response variable (*A*_*i*_) is strongly influenced by the chosen independent variables, as evidenced by the expected values closely clustered around the mean (Fig. 4A). The model demonstrated a strong fit to the data, as evidenced by a high pseudo R^2^ (0.78). The magnitude and direction of the environmental factors, which significantly influenced the *A*_*i*_, are reported in Table 4 and Fig. 4B. These results suggest that the environmental factors considered in this study are critical determinants of the differences in *Cx. pipiens* s.s. abundance found across different hibernacula. Temperature, humidity and light intensity showed a negative effect on *A*_*i*_, indicating that increases in these factors are associated with a decrease in the *Cx. pipiens* s.s. abundance. In particular, light has a stronger negative impact, as indicated by its large coefficient (–1.77), than humidity and temperature. Contrarily, VPD showed a positive association with *A*_*i*_. The relationships of light intensity vs. VPD and humidity vs. temperature are illustrated in Fig. 4C and D, respectively. Regarding the coefficients for the categorical variable hibernaculum category (Table 4), the building and natural cave categories showed negative estimates compared to the reference category, artificial cavity. The resulting odds ratios for buildings and natural caves were 0.025 and 0.036, respectively, indicating that their odds of *A*_*i*_ were only 2.5% and 3.6% of those for artificial cavities. The changes in *A*_*i*_ regarding temperature, humidity, light intensity and VPD for each hibernaculum category are reported in Additional file 1: Fig. S1.

**Table 4:** Results of the beta regression model, where artificial cavity was considered as the hibernaculum category of reference

Parameter	Estimate (β)	Standard error	z value	*P*-value
Intercept	18.286	1.033	17.707	< 0.01
Temperature (°C)	− 0.417	0.050	− 8.302	< 0.01
Humidity (%)	− 0.171	0.015	− 11.442	< 0.01
VPD (kPa)	2.567	1.003	2.559	0.01
Log_10_(Light + 1) (Lux)	− 1.767	0.032	− 54.567	< 0.01
Hibernaculum category: Building	− 3.696	0.084	− 43.919	< 0.01
Hibernaculum category: Natural cave	− 3.320	0.148	− 22.468	< 0.01

The model converged successfully after 15 iterations using the BFGS (Broyden-Fletcher-Goldfarb-Shanno) algorithm and an additional five iterations using Fisher scoring

### *Culex pipiens* biological form identification and RDA results

#### Biological forms

A total of 260 *Cx. pipiens* s.s. females, from eight overwintering sites, were analyzed and identified as follows, omitting samples that did not give clear results (*N* = 39): *Cx. pipiens pipiens* (*N* = 136), *Cx. pipiens molestus* (*N* = 40) and *Cx. pipiens pipiens/molestus* (*N* = 45). The sites were healthcare facilities (FI 1 and FI 2), abandoned country houses (LT 11), caves dug by humans as shelters for animals (LT 10 and LT 12) or to serve as cellars (LT 13), and two archeological sites (Etruscan tombs, RM 6, and Roman cisterns, LT 2). The sympatric occurrence of the two biological forms was highlighted in four hibernacula located in urban (FI 1) and rural areas (LT 2, LT 10 and LT 12), while hybrids were present at all sites.

#### Small-scale environmental variables

**Fig. 5:**
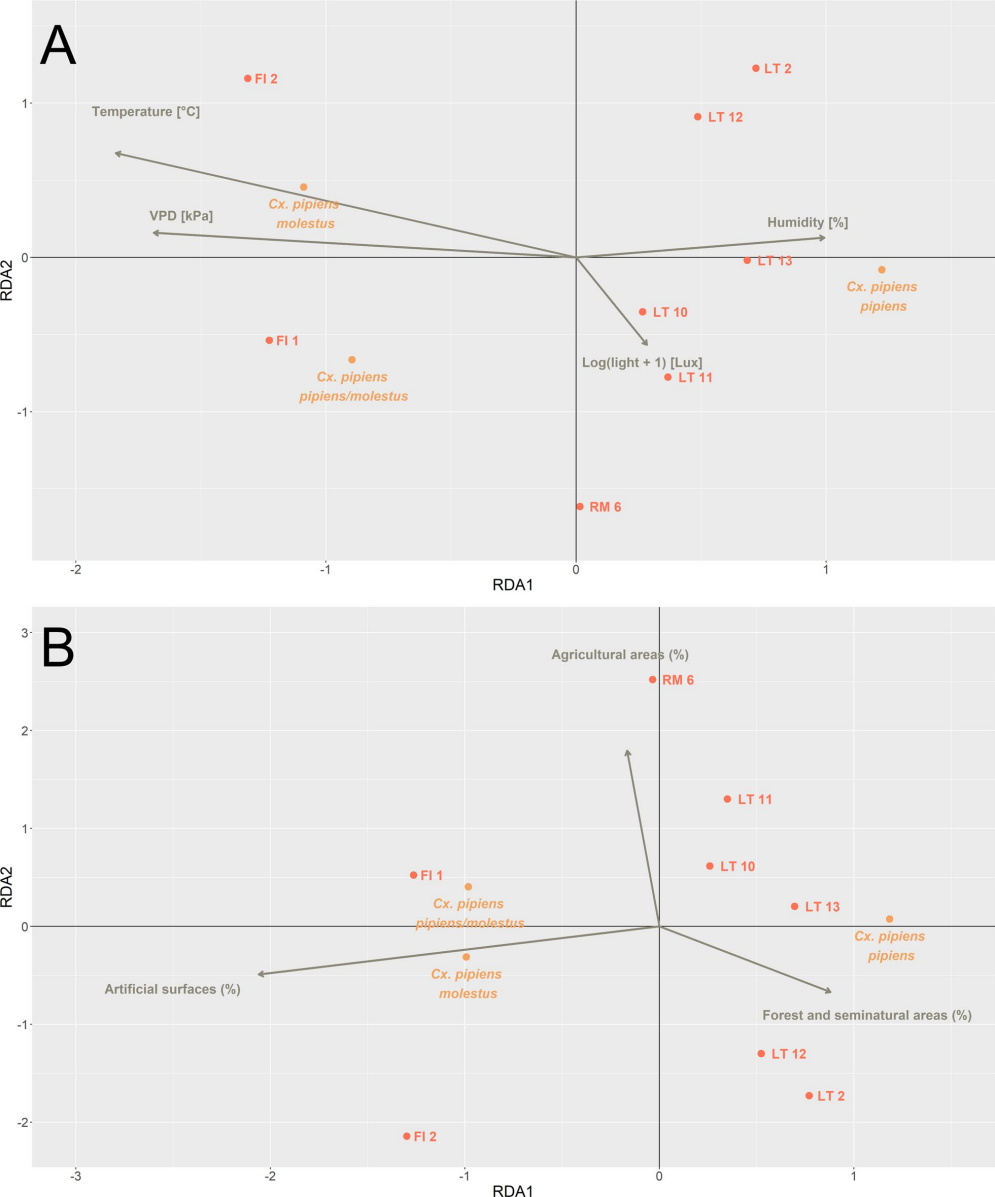
Results of the RDA models performed with small-scale environmental variables (**A**) and landscape variable (**B**) as predictors of the *Cukex pipiens* biological form composition. The plots show the relationship between environmental variables and the *Cx. pipiens pipiens*, *Cx. pipiens molestus* and *Cx. pipiens pipiens/molestus* abundance. To improve visualization, the length of the arrows representing the environmental variables was scaled using the scaling constant of RDA (score = 2.14). The red points correspond to sample sites, and orange points indicate the biological forms

**Fig. 6:**
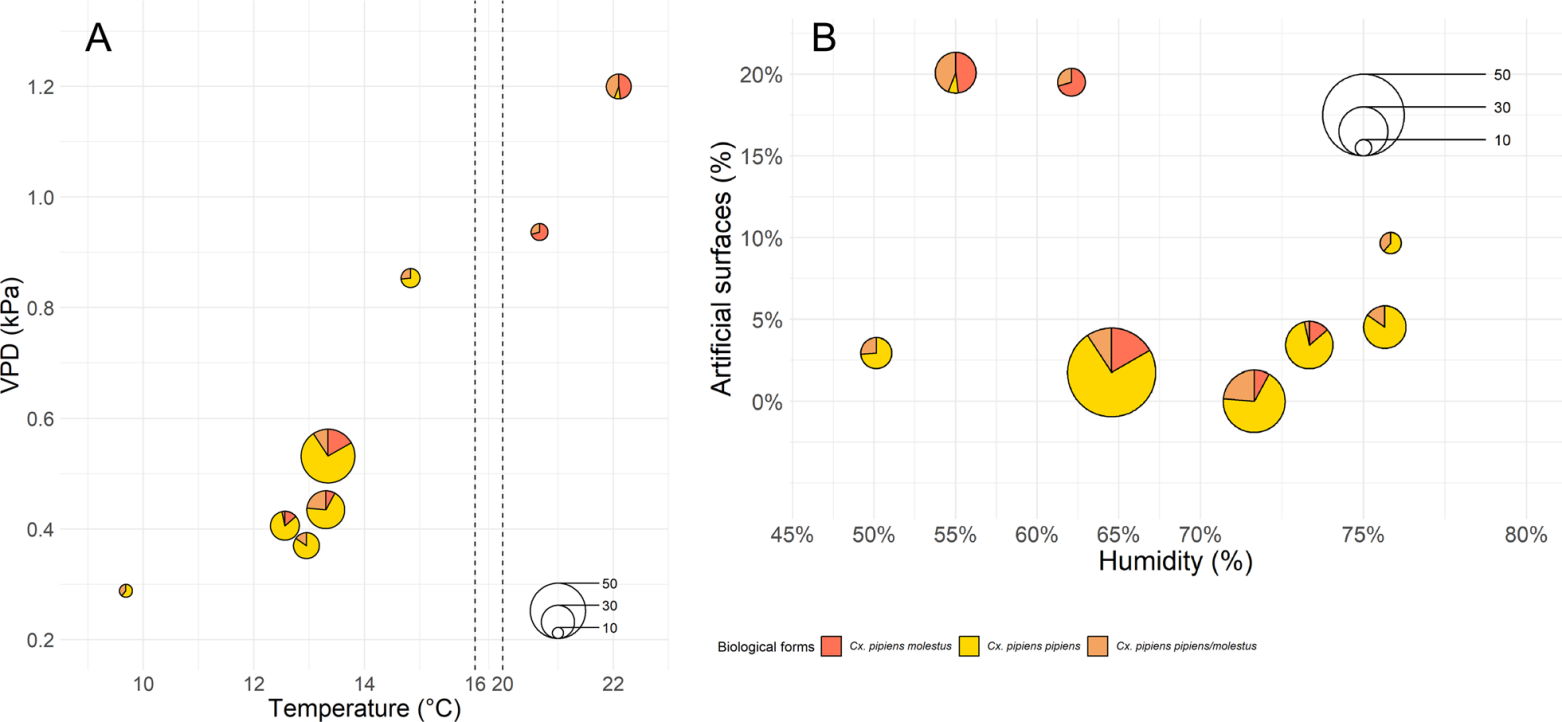
Scatterpie plots to show the relationship between the relative abundance of *Culex pipiens* biological forms and VPD vs. temperature (**A**) and humidity vs. artificial surfaces (%) (**B**). The scatterpie plot shows the percentage of *Cx. pipiens pipiens*, *Cx. pipiens molestus* and *Cx. pipiens pipiens/molestus* within the different hibernacula. The size of the pie charts represents the total number of individuals identified at each site, while the colors indicate the biological form. **A** Values between 16 and 20 °C were compressed to ease the visualization (dashed vertical lines). **B** Values > 70% were expanded, providing a clearer visualization of the data distribution at higher humidity levels

The results of the RDA performed with small-scale environmental variables as predictors indicated that the model accounted for 90.3% of the total variance in the relative abundance of the biological forms of *Cx. pipiens* within the inner rooms of the selected hibernacula (*N* = 8). The permutation-based ANOVA confirmed that the predictors significantly influenced the distribution of response variables (d.f. = 4, F = 6.99, *N* = 0.028). The first two RDA axes captured 76.0% and 14.3% of the total variance, respectively (Fig. 5A). Regarding the position of the *Cx. pipiens* biological form in the RDA space, the results showed a clear distinction between *Cx. pipiens pipiens* and *Cx. pipiens molestus*. *Culex pipiens molestus* was on the negative portion of RDA1 (score = − 1.08) and positive portion of RDA2 (score = 0.45), while *Cx. pipiens pipiens* was on the positive portion of RDA1 (score = 1.22) and on the negative portion of RDA2 (score = − 0.07). Hybrids *Cx. pipiens pipiens/molestus* presented a moderate negative association with both RDA1 (score = − 0.89) and RDA2 (score = − 0.66). Regarding environmental variables, temperature and VPD showed a negative association with RDA1 (score = − 0.85 and score = − 0.78, respectively) and a weak positive association with RDA2 (score = 0.31 and score = 0.07, respectively). Conversely, humidity exhibited a positive association with RDA1 (score = 0.46), while light intensity showed a lower positive association with RDA1 (score = 0.13). The scatterpie plots (Fig. 6) illustrate changes in relative abundance of biological forms regarding the main small-scale environmental variables, namely those strongly associated with RDA1. The *Cx. pipiens pipiens* biological form was observed at temperatures ranging from 10 to 15 °C and VPD values between 0.3 and 0.9 kPa (Fig. 6A). In contrast, the percentage of *Cx. pipiens molestus* decreased with increasing humidity levels (Fig. 6B). Hybrids were observed across a broader range of temperature, humidity and VPD, as shown in Fig. 6A and B.

#### Landscape-scale environmental variables

The results of the RDA performed using landscape variables (artificial surfaces, agricultural areas and forest and seminatural areas) as predictors indicated that the model accounted for 79.0% of the total variance in the relative abundance of *Cx. pipiens* biological forms across the selected sites (*N* = 8). The permutation-based ANOVA confirmed that these predictors significantly influenced the distribution of the response variables (d.f. = 3, F = 5.02, *P* = 0.02). The first two RDA axes captured 73.2% and 5.8% of the total variance, respectively (Fig. 5B). Regarding the positioning of *Cx. pipiens* biological forms in the RDA space, the results demonstrated a clear separation. Specifically, *Cx. pipiens pipiens* was positioned on the positive side of RDA1 (score = 1.18) and weakly associated with RDA2 (score = 0.07), while *Cx. pipiens molestus* and *Cx. pipiens pipiens/molestus* occupied the negative side of RDA1 (respectively score = –0.99 and score = –0.98). Concerning the environmental variables, artificial surfaces (%) were strongly and negatively associated with RDA1 (score = –0.96) (Fig. 5B), while forest and seminatural areas (%) had a moderate positive association with RDA1 (score = 0.41). The agricultural areas (%) did not contribute significantly to the distinction of the biological forms, as suggested by its association with RDA2 (score = 0.84). Given the significance of artificial surface (%) as an explanatory variable, the associated changes in the relative abundance of the biological forms of *Cx. pipiens* are shown in Fig. 6B.

## Discussion

The results presented in this study can be considered the first attempt to our knowledge to identify the overwintering sites of *Cx. pipiens* in Italy, aiming to highlight the small-scale environmental and landscape variables that are crucial for *Cx. pipiens* s.s. abundance. In addition, an in-depth molecular analysis was conducted to determine how the biological forms of *Cx. pipiens* respond to these factors.

Concerning results on the relative abundance of collected mosquitoes within the overwintering sites reported, *Cx. pipiens* s.s. was the most abundant species, with *An. maculipennis* s.l., *Cs. annulata* and *Cx. hortensis* accounting only for a lower proportion of the total catches. These findings accord with previous studies on hibernating mosquitoes in central and northern Europe [25–27, 43, 44]. Our results confirm that different types of shelters—from abandoned concrete structures to natural caves—fulfill the ecological requirements of this species, allowing adult females to enter diapause [45, 46]. The most abundant catches of *Cx. pipiens* s.s. were performed at sites located in rural areas, mainly belonging to the artificial cavity hibernaculum category. Two exceptions were an abandoned group of country houses (hibernaculum category: building) and a cave near Fondi Lake (hibernaculum category: natural cave). Indeed, the results of the beta regression model indicated that the artificial cavities might offer more favorable conditions than natural caves or buildings. Nevertheless, it is worth noting that the landscape around sampling sites was mainly characterized by agricultural areas or semi-natural habitats. Thus, the high *Cx. pipiens* abundance might be the consequence of the suitable habitat represented by rural areas [32]. Our findings also confirm that overwintering *Cx. pipiens* s.s. females can be found within both underground and aboveground sites [24, 25]. Indeed, it has been evidenced that underground structures represent suitable hibernacula for *Cx. pipiens* [46], as well as aboveground ones, especially if thermally insulated [25, 47]. Moreover, among the identified hibernacula, three were located in public parks in Rome, consisting of natural caves and an abandoned building.

Light intensity showed the most marked difference between entrances and inner rooms, and the reduction of brightness had the strongest positive influence on the *Cx. pipiens* s.l abundance within the inner room. These results align with previous studies on the importance of light conditions in overwintering site selection by *Cx. pipiens* s.s. [24, 48]. Indeed, it has been shown that prolonged exposure to light induces the cessation of hibernation by reactivating the metabolism of mosquitoes that start looking for a blood meal [49]. It could therefore be assumed that female mosquitoes might perceive complete and constant darkness as a cue of an isolated environment, subject to low fluctuations in temperature and humidity. This result is in line with recent research highlighting the crucial role of light, including artificial light, in influencing the physiological mechanisms associated with diapause in *Cx. pipiens*, altering its behavior and metabolism.[50].

Regarding humidity and VPD, two co-dependent environmental variables, their influence on *Cx. pipiens* overwintering ecology is controversial and has long remained a debated issue. Owen (1937) reported the presence of millions of *Cx. pipiens* in a mushroom cave, a markedly moist environment. This observation has been reported by many authors [51, 52] and was confirmed in a work conducted in Sweden, where overwintering *Cx. pipiens* were collected in a cellar with > 90% humidity [43], reinforcing the idea that high humidity plays an important role in the overwintering of this species. Further studies carried out on overwintering mosquitoes concluded that humidity was not a determining factor for *Cx. pipiens* mortality or diapause site selection [24, 25]. Based on our results, it can be assumed that the species has a wide tolerance range for humidity levels, in line with previous findings [46]. Particularly, even though the highest abundance of *Cx. pipiens* s.s. was recorded in the inner rooms, characterized by high humidity and low VPD, among the darker rooms, mosquitoes seemed to prefer less humid ones (at higher VPD). Indeed, the beta regression results presented a negative correlation between *Cx.pipiens* s.s. abundance and humidity, as previously reported [53]. Seeking low humidity and high VPD suggests that these mosquitoes could adapt to dry conditions during their overwintering phase. Indeed, overwintering *Cx. pipiens* adults are considerably more tolerant of desiccation than their nondiapausing counterparts [54]. Adaptation to low levels of air moisture may be a consequence of the proven mortality of *Cx. pipiens* due to entomopathogenic fungi, whose growth is hindered in dry environments [55–57].

Beta regression results indicated that the decrease in mean temperatures in the inner rooms correlated with an increase in *Cx. pipiens* s.s. abundance. These results provide field evidence of the importance of temperature for diapausing success of *Cx. pipiens* s.s. females, confirming a previous laboratory investigation [25]. Indeed, it is well known that high temperatures, by increasing mosquitoes’ metabolic rates, deplete their lipid reserves, forcing females to leave their hibernacula and seek energy sources [48, 58]. Overwintering *Cx. pipiens* were more tolerant of low temperatures than nondiapausing ones [59], even though testing of natural and laboratory populations has highlighted limits to *Cx. pipiens* cold hardiness (e.g. < 5 days at − 5 °C [59]; e.g. < 7 days at − 4 °C [60]). Nevertheless, in our study, the temperature of the hibernacula never dropped below these limits, allowing for safe winter survival even in aboveground shelters, considered less suitable in central Europe [60]. On the other hand, in heated environments—such as the hospital and clinic—active *Cx. pipiens* adults and a larval breeding site, with several larvae and pupae, were found. These results indicate that at high temperatures and with accessible blood meals, homodynamic *Cx. pipiens* populations could be observed.

These results revealed that the two biological forms of *Cx. pipiens* coexist in overwintering sites in central Italy. In particular, the presence of at least one biological form of *Cx. pipiens*, alongside hybrids, was noted at all the sites analyzed, while 50% of the sites sheltered both *Cx. pipiens pipiens* and *Cx. pipiens molestus*, consistent with a previous report [61] confirming the pattern whereby in southern Europe and northern Africa hybridization events are frequent and the two biological forms are able to colonize the same habitat [16, 53, 61–63]. In contrast, in central and northern Europe, hybrids occur at much lower rates than those reported in our study (e.g. 6–15% in The Netherlands [64]), probably because of differential habitat selection of the two biological forms [12, 14, 65]. Interestingly, at two healthcare facilities, *Cx. pipiens molestus* was found in association with a high percentage of hybrids, and at one of them a larval breeding site was identified in a flooded basement (Fig. 2F), similarly to what was described by Di Luca et al. [63]. Compelling evidence confirms that environmental changes due to human activities allow *Cx. pipiens* mosquitoes, particularly the biological form *molestus*, to find suitable sites for larval development during winter [12]. Besides, this finding indicates that the hybrids could be homodynamic, behaving like *Cx. pipiens molestus*. Within hibernacula located in rural areas, characterized by lower temperatures than in the aforementioned health facilities, even if *Cx. pipiens molestus* was present, mosquitoes showed no signs of activity (e.g. larval breeding sites were absent and mosquitoes rested almost motionless, attached to vertical surfaces). This observation confirms the previous hypothesis that, depending on environmental conditions, *Cx. pipiens molestus* can express or suppress diapause [46, 66]. The hybrids were present in a variety of overwintering sites, ranging from abandoned or used buildings to caves dug into the rock in ancient or modern times, including two archeological sites, demonstrating a high capacity to occupy heterogeneous habitats. This outcome has epidemiological relevance, as hybrids may favor the transmission of WNV from birds to humans due to their intermediate feeding behavior between the anthropophilic *molestus* and ornithophilic *pipiens* forms [67, 68]. However, it is crucial to consider the relative and absolute abundance of the biological forms of *Cx. pipiens* and hybrids when assessing the risk of WNV transmission. Although hybrids have lower transmission rates than the *pipiens* biological form, they exceed those of *molestus* and increase with increasing temperatures [69], suggesting a higher risk of transmission in populations dominated by *pipiens* and hybrids.

Even though the two biological forms co-occurred within half of the analyzed hibernacula, the results of the RDA models suggested that selected environmental variables, at both the small and landscape scale, might inform the relative abundance of the biological forms of *Cx. pipiens*. In particular, *Cx. pipiens pipiens* and *Cx. pipiens molestus* showed opposite responses to changes in temperature, humidity, VPD and the transition from natural to anthropogenic environments. The biological form *molestus* was observed in warmer sites, characterized by lower humidity levels and higher VPD, while the biological form *pipiens* was prevalent within shelters with lower temperatures, higher humidity levels and lower VPD. Considering landscape variables, *Cx. pipiens pipiens* was primarily associated with forested and seminatural areas, whereas *Cx. pipiens molestus* was more frequently found at sites with a high proportion of artificial surfaces. Furthermore, light intensity and percentage of agricultural area were not associated with the relative abundance of the two biological forms, suggesting that darkness may be an important factor for both forms of *Cx. pipiens* and that both colonize rural environments. These could be a consequence of the tendency of *Cx. pipiens molestus* to occupy human-made underground shelters during winter, which, combined with its pronounced anthropophilic feeding behavior, led it to colonize warmer microclimates [44, 64]. In fact, our results confirm previous findings on the preference of *molestus* for anthropogenic habitats [16, 65, 70, 71] while being consistent with the ubiquitous distribution of the biological form *pipiens* [61]. Moreover, the presence of the biological form *pipiens* has also been recorded in subterranean hibernacula, historically considered to be favored by *Cx. pipiens molestus* [67, 72].

The following findings—(i) the co-occurrence of the two biological forms (in rural or anthropogenic environments, within above- and underground shelters), (ii) the widespread presence of hybrids and (iii) the ability of *Cx. pipiens* form *molestus* to express or suppress diapause—reflect a remarkable phenotypic plasticity within the species. Thus, although differential life history traits, which may have evolved as adaptive mechanisms to distinct environments, might be used to predict the relative abundance of the two biological forms, a certain degree of phenotypic plasticity may still allow overlap in habitat selection.

## Conclusions

This study provides a detailed description of *Cx. pipiens* overwintering sites with in-depth analyses of the environmental conditions. The epidemiological significance of this research lies in the role played by *Cx. pipiens* biological forms and hybrids in the circulation of WNV and USUV. In particular, the presence of hybrids in anthropogenic environments, with a notable abundance in healthcare facilities, combined with the species’ anthropophilic feeding strategy [73], raises significant public health concerns. In addition, overwintering *Cx. pipiens* can not only act as an arbovirus reservoir [43] but, especially in Mediterranean countries, also remain active during mild winters, enabling continuous transmission of WNV [74]. Our results, together with previous knowledge, highlight the importance of extensive mosquito surveillance and monitoring, aimed at both the detection of hibernacula and their possible management. In general, the abundant catches performed at several overwintering sites suggest the hypothesis of vector control measures targeted at *Cx. pipiens* females within hibernacula. This strategy may not only reduce the mosquito population of the following spring [29, 75] but also limit the disease burden that this vector could pose during the following transmission season [76].

## Supplementary Information

Additional file 1.

Additional file 2.

## Data Availability

The data that support the findings of this study are available from the corresponding author upon reasonable request.

## Abbreviations

WNV: West Nile virus
VPD: Vapor pressure deficit
A_i_: Abundance index
RDA: Redundancy analyses
CLC: Corine Land Cover
