# Supplementary material for: Characterization of overwintering sites (hibernacula) of the West Nile vector Culex pipiens in Central Italy
Source: Parasit Vectors. 2025 Feb 24;18:74. doi: 10.1186/s13071-025-06710-5 (PMC11852880; doi:10.1186/s13071-025-06710-5)
Supplement: Supplementary file 2 — Additional file 2. [file 13071_2025_6710_MOESM2_ESM.docx]

**Supplementary information**

**Additional file 1: Fig. S1.** Effect of mean temperature (A), humidity (B), VPD (C), light (D) on the abundance index (*Ai*) of *Culex pipiens* s.l. across different hibernaculum types, based on beta regression model. The lines represent predicted values from the beta model for each hibernaculum category: artificial cavities (grey), buildings (black), and natural caves (dark orange). The shaded bands around each line indicate 95% confidence intervals.
